# Mapping temperate old-growth forests in Central Europe using ALS and Sentinel-2A multispectral data

**DOI:** 10.1007/s10661-024-12993-5

**Published:** 2024-08-26

**Authors:** Devara P. Adiningrat, Michael Schlund, Andrew K. Skidmore, Haidi Abdullah, Tiejun Wang, Marco Heurich

**Affiliations:** 1 Faculty of Geo-Information Science and Earth Observation, University of Twente, Enschede, The Netherlands; 2 Department of National Pak Monitoring and Animal Management, Bavarian Forest National Park, Grafenau, Germany; 3 Chair of Wildlife Ecology and Wildlife Management, University of Freiburg, Freiburg, Germany; 4 Institute for Forest and Wildlife Management, Inland Norway University of Applied Science, Koppang, Norway

**Keywords:** Airborne LiDAR, Multispectral, Data fusion, Forest structure, Stand age, Structural complexity

## Abstract

Old-growth forests are essential to preserve biodiversity and play an important role in sequestering carbon and mitigating climate change. However, their existence across Europe is vulnerable due to the scarcity of their distribution, logging, and environmental threats. Therefore, providing the current status of old-growth forests across Europe is essential to aiding informed conservation efforts and sustainable forest management. Remote sensing techniques have proven effective for mapping and monitoring forests over large areas. However, relying solely on remote sensing spectral or structural information cannot capture comprehensive horizontal and vertical structure complexity profiles associated with old-growth forest characteristics. To overcome this issue, we combined spectral information from Sentinel-2A multispectral imagery with 3D structural information from high-density point clouds of airborne laser scanning (ALS) imagery to map old-growth forests over an extended area. Four features from the ALS data and fifteen from Sentinel-2A comprising raw band (spectral reflectance), vegetation indices (VIs), and texture were selected to create three datasets used in the classification process using the random forest algorithm. The results demonstrated that combining ALS and Sentinel-2A features improved the classification performance and yielded the highest accuracy for old-growth class, with an F1-score of 92% and producer’s and user’s accuracies of 93% and 90%, respectively. The findings suggest that features from ALS and Sentinel-2A data sensitive to forest structure are essential for identifying old-growth forests. Integrating open-access satellite imageries, such as Sentinel-2A and ALS data, can benefit forest managers, stakeholders, and conservationists in monitoring old-growth forest preservation across a broader spatial extent.

**Supplementary Information:**

The online version contains supplementary material available at 10.1007/s10661-024-12993-5.

## Introduction

Old-growth forests are forest stands that reach the ultimate stage of stand development characterized by old and large trees, multi-layered structures, and deadwood abundance with limited human activities (O’Brien et al., 2021; Wirth et al., 2009). Old-growth forests can also be developed under human intervention through a strict or passive management strategy that conserves the old-growth attributes (de Assis Barros & Elkin, 2021; van der Knaap et al., 2020). These forests are essential for providing ecosystem services, such as maintaining biodiversity (Hilmers et al., 2018; O’Brien et al., 2021), storing carbon (Luyssaert et al., 2008), and mediating microclimate (Frey et al., 2016). Although mandated in the EU Biodiversity 2030 strategy as a priority for protection, old-growth forests in Europe are threatened and increasingly scarce due to logging and land conversion (Hirschmugl et al., 2023; Mikoláš et al., 2023; O’Brien et al., 2021). Old-growth forests in Europe are also vulnerable to insect outbreaks due to the impact of the rising temperature driven by climate change (Forzieri et al., 2021). Therefore, detailed mapping of the extent and distribution of old-growth forests in Europe is needed to support improved conservation management strategies for these at-risk ecosystems (Hirschmugl et al., 2023).

Efforts have been made using many approaches to map the distribution of the old-growth forests, from ground observations to remote sensing (Barredo et al., 2021; Hirschmugl et al., 2023). While field observations remain critical to capturing certain attributes of old-growth forests, recent research has demonstrated the ability of state-of-the-art remote sensing technologies for inventorying and monitoring old-growth forests (Hirschmugl et al., 2023). Remote sensing has a significant role in biodiversity mapping, specifically for old-growth forests, given its advantage of covering large, continuous areas with a synoptic overview (Hirschmugl et al., 2023; Skidmore et al., 2021). Often, the remaining old-growth forests in Europe are located in remote areas, which are difficult to access with field surveys, and here, remote sensing is central to obtaining information from such locations (Hirschmugl et al., 2023; Spracklen & Spracklen, 2019). For these remote locations, remote sensing can be particularly efficient and less labor-intensive than field surveys for identifying old-growth forest indicators (Spracklen & Spracklen, 2019).

Multispectral remote sensing has been utilized for mapping forest stand developments long before laser scanning technology became a trend in the late 1990s. The mapping mainly rely on spectral variability of different forest structure developments in old-growth and younger stages (Cohen & Spies, 1992; Congalton et al., 1993; Fiorella & Ripple, 1993; Spracklen & Spracklen, 2019). This variability is mainly associated with changes in the horizontal dimensions, such as canopy cover, various tree proportions, and the emergence of shadows as an impact of tree density alteration (Scarth et al., 2001). Besides spectral reflectance, other multispectral features and approaches, such as image texture (Coburn & Roberts, 2004; Franklin et al., 2000; Spracklen & Spracklen, 2019; Zhang et al., 2017) and a combination with ancillary data, such as historical land cover/land maps, forest inventory attributes, and terrain attributes (elevation, slope, aspect) (Congalton et al., 1993; Spracklen & Spracklen, 2019), have been also optimized for old-growth forest mapping. These previous studies mainly utilized medium to high spatial resolution imageries from different platforms, such as satellite-based imagery, as a source for multispectral data.

Sentinel-2A is a publicly available multispectral satellite imagery launched under the European Copernicus program. Sentinel-2A provides an image with a higher spatial resolution (10 to 20 m) and denser time revisit (5 days) compared to other freely accessible imagery, such as Landsat (spatial resolution of 30 m with time revisit of 16 days) (Immitzer et al., 2016). Sentinel-2A imagery advances forest classification studies by offering great opportunities for a higher spatial and temporal resolution to ensure detailed forest ecosystem information (Immitzer et al., 2016; Spracklen & Spracklen, 2019). Nevertheless, relying on spectral reflectance from standalone multispectral imagery, such as Sentinel-2A, may be insufficient for old-growth forest assessment because multispectral imagery has limitations on the extraction of some of the forest structural attributes associated with the old-growth stage, such as vertical layering and detailed tree size (height and diameter) (Scarth et al., 2001). This is true for the old-growth stage, as it is mainly identified by its structural complexity comprised of canopy heterogeneity and multilayer/vertical profile, in which forest 3D information has a significant role (Lahssini et al., 2022; LaRue et al., 2019).

Light detection and ranging (LiDAR) is an active sensor with less signal saturation than the multispectral sensor and can penetrate multi-layered canopies, making it suitable for characterizing forest structures. Numerous studies have used an airborne laser scanner (ALS) as a platform to mount the LiDAR sensor. This technology has been used to derive detailed forest structure information from the top canopy to the ground, providing 3D forest estimation (Lahssini et al., 2022; Lefsky et al., 1999). With these advantages, ALS is often used for assessing, inventorying, and monitoring forest structures and stand development at the landscape scale, with an area-based approach (ABA) as the typical method to analyze the data (Fuhr et al., 2022; Lahssini et al., 2022; White et al., 2016). Area-based approaches calculate the spatial distribution of point clouds to generate grid features using statistical analysis. While ALS has many advantages for measuring forest structures, limitations to these datasets include inadequacy in penetrating sub-canopy and capturing understory information, relatively limited coverage, high cost per unit area sensed, and often being publicly unavailable (Falkowski et al., 2009; Hamraz et al., 2017; Hirschmugl et al., 2023; Spracklen & Spracklen, 2019).

Here, we propose a combination of structural information from ALS and spectral characteristics from Sentinel-2A to complement each sensor in delivering comprehensive old-growth forest mapping, particularly in capturing 3D forest structure complexity. Lahssini et al. (2022) and Scarth et al. (2001) demonstrated that combining multispectral and ALS data improves the characterization of forest structural complexity. ALS enhances 3D forest assessment, while Sentinel-2A excels in identifying horizontal changes (i.e., canopy cover, shadow, and stand composition) over vast areas, facilitating comprehensive estimation of structural information in extensive old-growth forest coverage. To our knowledge, the use of a combination of ALS and optical data for old-growth forest mapping over large areas in Central European mixed temperate forest ecoregion is still limited. The approach of using remote sensing for mapping old-growth forests at a large spatial extent was more common in North American temperate forests and only recently applied in the Carpathian ecoregion in Europe (Hirschmugl et al., 2023; Spracklen & Spracklen, 2019). Therefore, our study provides insights and contributes to the development of mapping approaches and management of old-growth forests within the ecoregion of Central European mixed temperate forests.

We aimed to investigate the combination of different Sentinel-2A multispectral features, i.e., reflectance, spectral indices, and texture data, with ALS data to optimize the old- and second-growth forest mapping in European temperate forests. Through this study, we addressed two objectives:

- To compare the classification accuracy improvements for old-growth forests using Sentinel-2A, ALS, and a combination of Sentinel-2A + ALS.
- To explore important ALS and Sentinel-2A features in identifying old-growth forests.

## Material and methods

### Study area

**Fig. 1:**
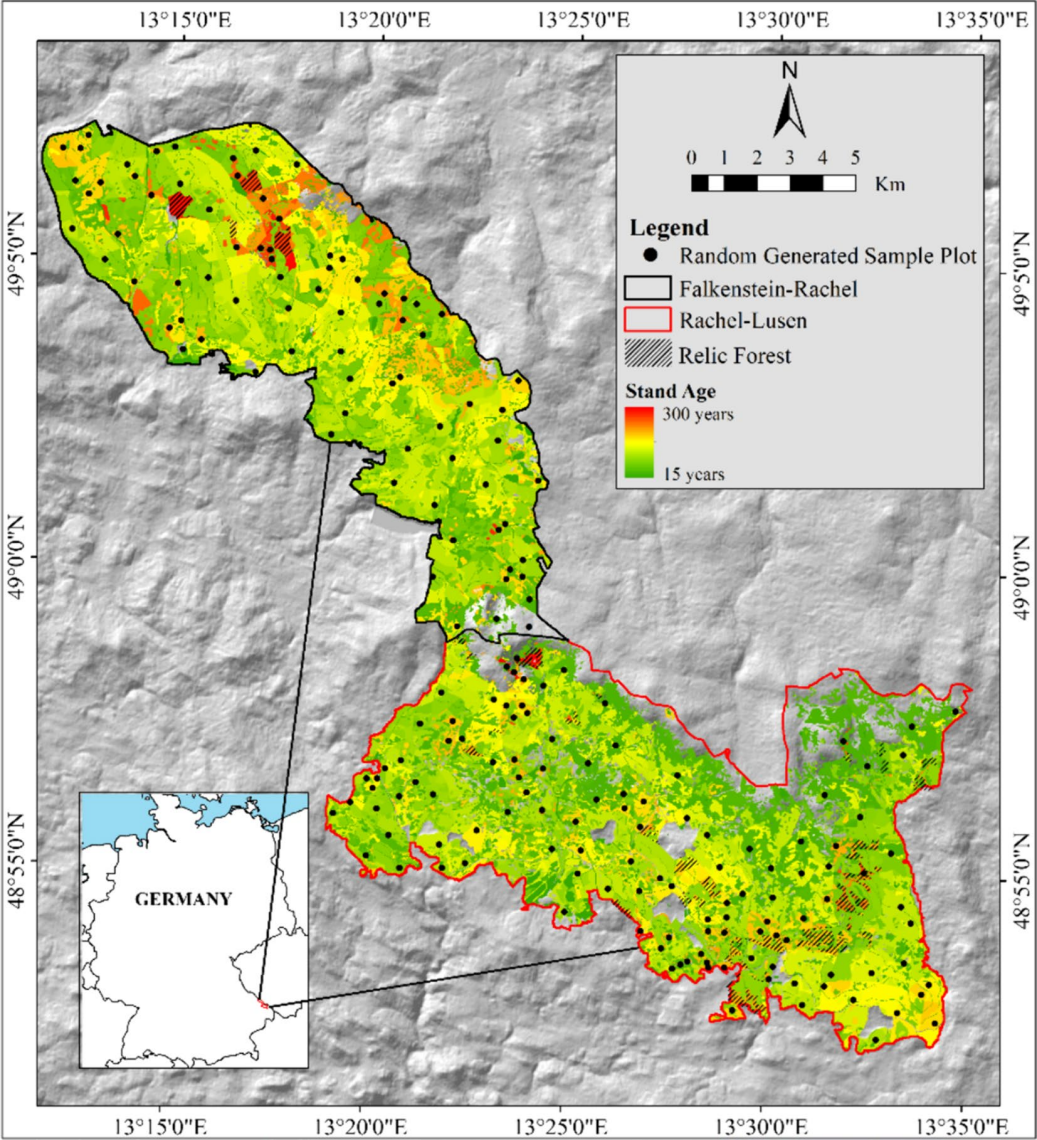
Map of Bavarian Forest National Park (BFNP) with the distribution of sample plots, which were randomly generated from the reference data of this study, i.e., BFNP stand age map. The random selection of plots was based on an old-growth stand age threshold of more than 150 years. Plots below 150 years were assigned as second-growth

The study was conducted in Bavarian Forest National Park (hereafter BFNP), which is situated in the southeastern part of Germany (43.055° N, 13.203° E) (Fig. 1). The BFNP is the oldest national park in Germany, and it preserves a mixed temperate forest landscape and is a part of the larger Bohemian Forest. It covers an area of 249 km^2^ with an altitude of 600 to 1453 m above sea level (Cailleret et al., 2014). The predominant species in BFNP is Norway spruce (*Picea abies*), which concurrently inhabits the slopes with European beech (*Fagus sylvatica*) and silver fir (*Abies alba*) at the lower elevations (Cailleret et al., 2014; Heurich & Englmaier, 2010).

The park has a long history of forest management and natural disturbance since the seventeenth century (Heurich & Englmaier, 2010). It was established as a national park in 1970 in the Rachel-Lusen area (the southern part), with the northern part of BFNP (Falkenstein-Rachel area) included in 1997 (van der Knaap et al., 2020). The disturbance that occurred in BFNP from the seventeenth century onwards included windthrow, snow breakage, and bark beetle infestations, with forestry operations mainly focused in the Rachel-Lusen area (Heurich & Englmaier, 2010). There was a substantial decrease in forest management in the Rachel-Lusen area during the 1980s following the creation of management zones confining forestry activities. Active forest management persisted in the Falkenstein-Rachel area, with the non-intervention zone (natural zone) gradually expanding since its establishment in 1997. Since then, the park has gradually increased the non-intervention area (van der Knaap et al., 2020). According to the land cover map of BFNP (Silveyra Gonzalez et al., 2018), nowadays, 50% area of BFNP is covered by forested areas, of which 87% is dominated by mature and potentially old-growth stands and 13% by earlier-stage forests. These contemporary proportions are the result of the non-intervention management strategy in the park. Further, approximately 250 ha have been identified by park management as “relic forests” which have been managed without any intervention for at least 250 years (Heurich & Englmaier, 2010) (see the locations in Fig. 1). The historical development of the BFNP forests suggests that mature and old forest stands (ranging from 50 to over 300 years old) dominate the landscape (Moning & Müller, 2009).

### Determining stand age from reference stand age map

We used existing information from the BFNP stand age map as a reference to determine the old-growth forest areas. The stand age map was generated from an interpolation of permanent forest inventory plots with a size of 0.05 ha (Moning & Müller, 2009). There were 2000 inventory plots distributed across BFNP and placed using systematic (grid) sampling with a 200-m distance between the plots to ensure all forest conditions within BFNP were captured. After the age of trees was measured, a generalization process integrated plots of similar age into a stand age polygon map with a scale of 1:50.000. In Rachel-Lusen, the stand age determination was executed by tree rings measurement using tree coring. In contrast to Rachel-Lusen, the stand age determination in Falkenstein was conducted by estimating the tree rings of a stump and then comparing it with the surrounding trees of a similar size. This approach assumes that the stump is associated with the surrounding trees when they have similar sizes and, therefore, similar ages. All the measurements were conducted in 1986 (Falkenstein-Rachel) and 1991 (Rachel-Lusen), and each year, the park management updates the database by adding 1 year of age to each stand age polygon. As the latest update of the used stand age map was in 2002, we added 15 years for each stand age polygon to better estimate the stand age with the remote sensing data acquisition in mid-2017. During this period, no, or eminently limited, active forest management was undertaken within the study area.

**Fig. 2:**
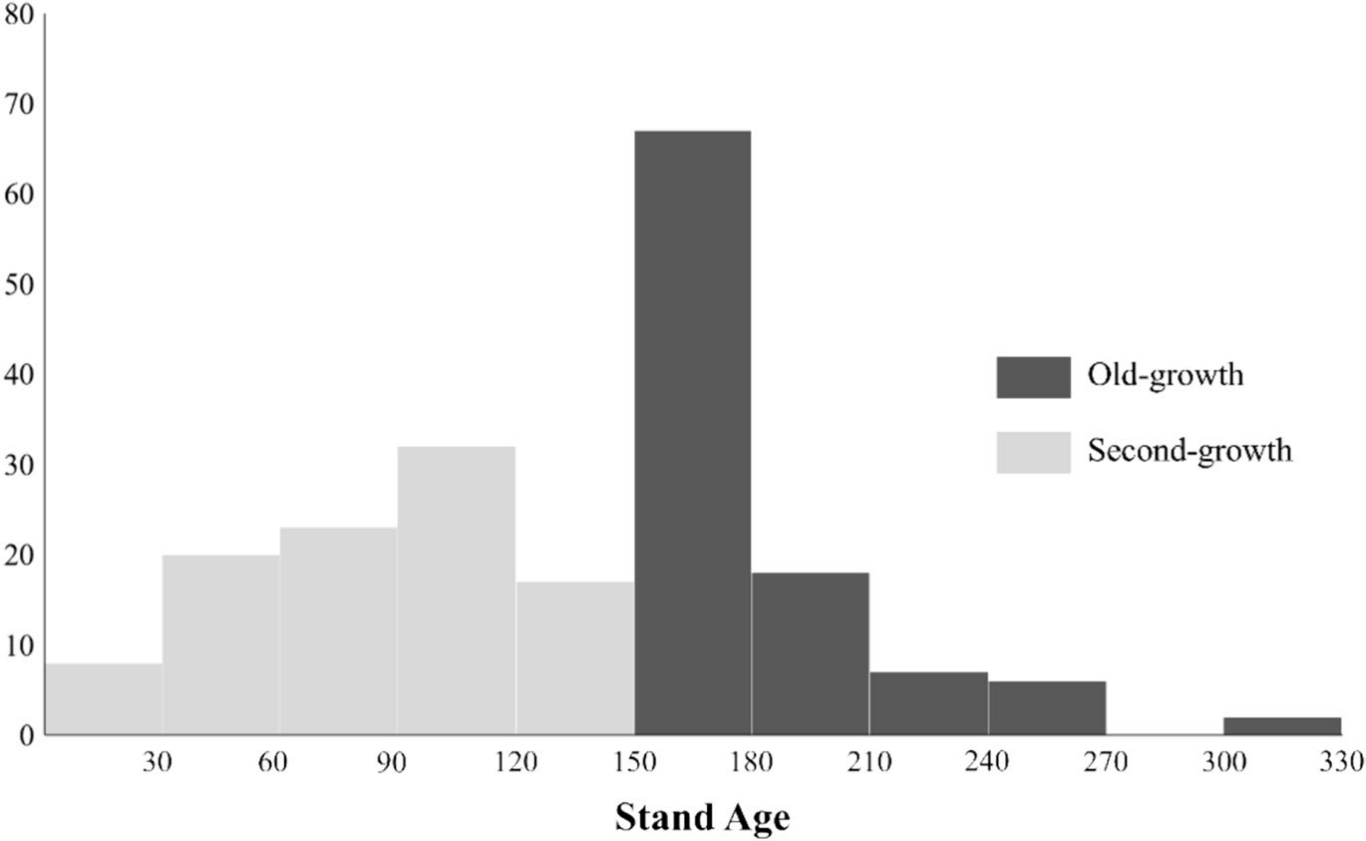
The frequency of the stand age of the old-growth and second-growth plots of this study

We referred to stand development classes developed by Oliver and Larson (1996) using a threshold of more than 150 years old to assign an old-growth forest area. The threshold of 150 years has also been widely used to define the old-growth stand in European temperate forests (Brunet et al., 2010; Vandekerkhove et al., 2022; Wirth et al., 2009). Piovesan and Biondi (2021) propose that an old-growth stand is characterized by the average age of dominant trees surpassing half of their respective lifespans. In Europe (including BFNP), Norway spruce and European beech typically exhibit a lifespan of around 300 years (San-Miguel-Ayanz et al., 2016). Hence, forest stands where Norway spruce and European beech dominate, such as in BFNP, are considered to enter an old-growth phase upon reaching the age of 150 years (Vandekerkhove et al., 2022). Forest stands with a threshold greater than 150 years were also found to demonstrate the old-growth characteristics such as self-thinning and tree mortality events that give complexity to forest structure (Donato et al., 2012; Franklin & Van Pelt, 2004; Franklin et al., 1981; Oliver & Larson, 1996). Additionally, some parts of BFNP consist of relic forests with an average stand age of about 170 years based on the stand age map. Therefore, we assumed that the 150-year threshold is reasonable for defining old-growth stands in BFNP within a European context. In addition, a stand age less than 150 years old in this study was assigned as a second-growth forest class. Generally, our old-growth plots were dominated by stand age within the range of 150 to 180 years old, while the second-growth plots were relatively dominated by stand age of 50 to < 150 years old (Fig. 2).

### ALS data processing and feature derivation

ALS data was acquired by Riegl LMSQ 680i LiDAR sensor with a wavelength of 1550 nm and a beam divergence of 0.5 mrad. The flight campaign was conducted in June 2017 and flown at an altitude of approximately 550 m under the leaf-on condition over the entire BFNP. Sixty percent of strip overlaps produced an average point cloud density of 30 to 70 points/m^2^ in the overlap tiles (Krzystek et al., 2020).

**Fig. 3:**
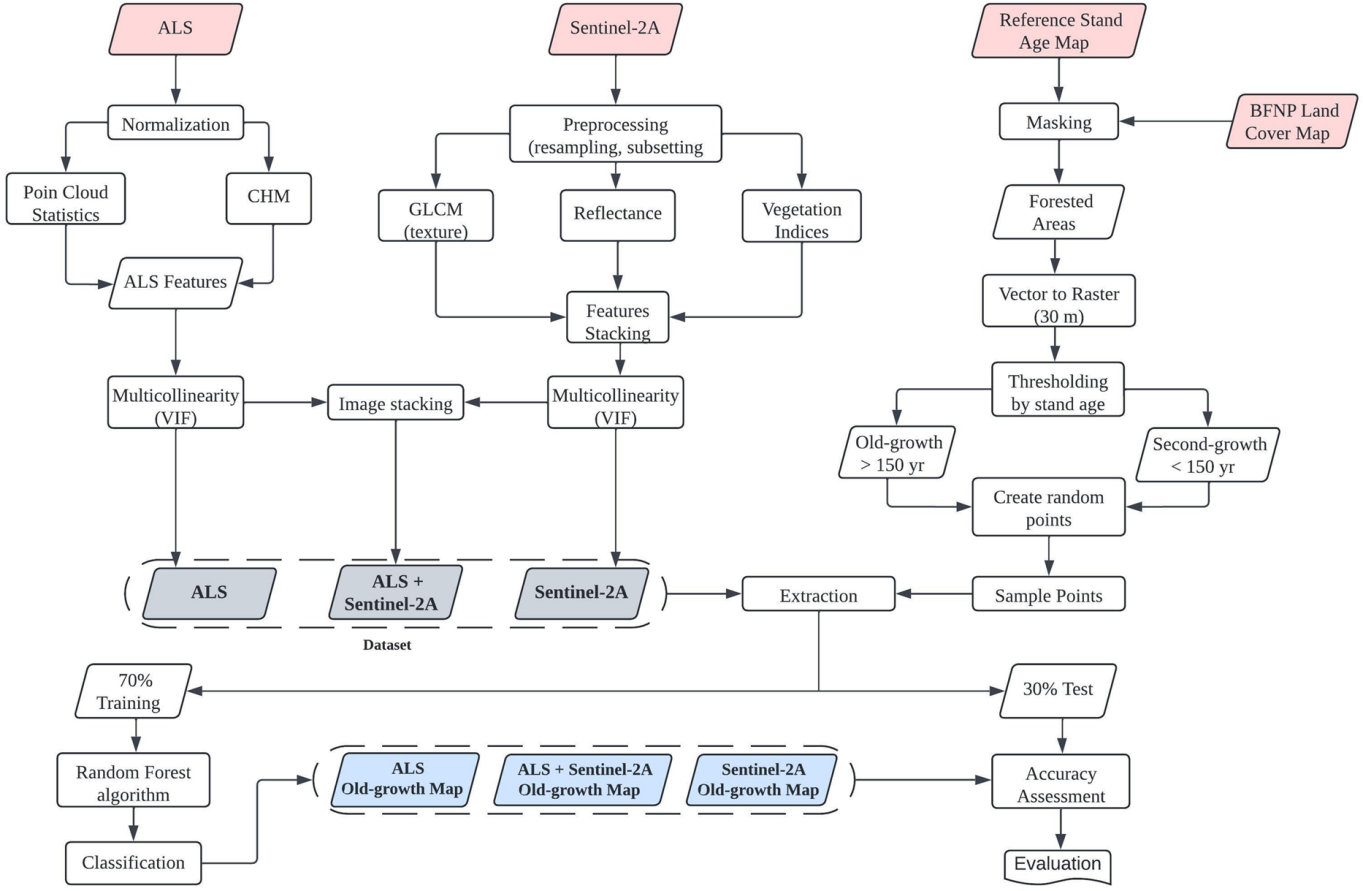
Workflow process used for this study. The workflow includes preprocessing ALS and Sentinel-2A data, generating sample plots from reference datasets, classifying datasets, and evaluating accuracy assessment and important features

The laser returns and height were normalized by interpolating the ground points to the position of below non-ground return (Fig. 3). A cloth simulation function (Zhang et al., 2016) with a cloth resolution of 1 m was used to classify the ground and non-ground points. The ground points were then interpolated using a triangle irregular network (Roussel et al., 2020). A canopy height model (CHM) was generated in 1-m resolution as some LiDAR features had to be generated by CHM. All the ALS features were gridded to 30-m resolution following the common medium spatial resolution of multispectral data and field plot dimension. The normalization and ground classification steps were conducted using the *lidR* package (Roussel et al., 2020) in R software version 4.1.2 (https://www.r-project.org/).

For the purpose of this study, 61 ALS features were generated and divided into two groups, i.e., standard and structural features (see Table S1 in the supplementary). Standard features are directly generated from the statistics of the point cloud profile and related to height distributions and the number of returns (Ayrey et al., 2019; Pearse et al., 2019). Structural features relate to the canopy’s horizontal and vertical profile complexity and require more advanced processes by incorporating CHM to generate the features (Atkins et al., 2018; LaRue et al., 2019). Multicollinearity analysis using the variation inflation factor (VIF) was applied to select the ALS features with low collinearity. We set a criterion of VIF ≤ 5 to ensure a more stringent collinearity threshold as the commonly used VIF ≤ 10 is argued as too lenient and distorts model estimation and accuracy (Hair et al., 2018). Four ALS features were selected based on the VIF analysis result (Table 1).

**Table 1:** Selected ALS features used in the classification. The selection was based on the multicollinearity analysis using the VIF approach. Rumple Index and VCI are from the structural features group, and zsd and zq85 are from the standard features group. All selected features have VIF ≤ 5

ALS features	Description
Rumple Index	Ratio between outer canopy surface area and projected ground surface
Vertical Complexity Index (VCI)	The distribution evenness of point cloud within a vertical layer
zsd	Standard deviation of height distribution
zq85	85th percentile of the height distribution

### Sentinel-2A data processing and feature derivation

Three feature groups of multispectral data were derived from Sentinel-2A imagery, i.e., raw bands (reflectance), vegetation indices (VIs), and texture (Fig. 3). The Sentinel-2A imagery was downloaded from ESA-Copernicus (https://scihub.copernicus.eu) at level 2A product where atmospheric (bottom-of-atmosphere) and terrain corrections had been applied to the image. The acquisition date is 13 July 2017 (the same period as ALS data acquisition) on leaf-on condition with 0% cloud cover in our study area. We performed the same approach with the ALS feature selection by applying VIF analysis to select the features of Sentinel-2A with low collinearity (VIF ≤ 5). All features (raw bands, VIs, and texture) were stacked prior to the VIF analysis, and the collinearity analysis was conducted on all features altogether (see Fig. 3). Based on the VIF result, 15 features were selected comprising three raw bands, two VIs, and ten textural images (see Table 5).

#### Raw band (reflectance) feature

Sentinel-2A has 13 bands with different spatial resolutions (10 m, 20 m, and 60 m) (Immitzer et al., 2016), with the ten bands used for this study comprised of four 10-m resolution bands and six 20-m resolution bands. The spectral resolution of Sentinel-2A is higher than previous satellite multispectral imageries, especially in the region between red to near-infrared (NIR) wavelengths with three additional red-edge bands (Band 5, Band 6, and Band 7), which are useful for vegetation studies and mapping (Frampton et al., 2013; Immitzer et al., 2016; Kiala et al., 2019). All bands were resampled using the nearest neighbor method to 30-m resolution following the ALS resolution. After the resampling, all bands were stacked and clipped into the area of interest.

#### Vegetation indices (VIs) feature

Four vegetation indices (VIs) were used in this study (Table 2). Two indices, i.e., the Normalized Difference Vegetation Index (NDVI) and the Enhanced Vegetation Index (EVI ), were used as they are the most common indices used in forest studies, especially for forest growth identification (Gao et al., 2023; Spracklen & Spracklen, 2019). Since the canopy gap is one of the indicators of old-growth forests, the Modified Soil-Adjusted Vegetation Index (MSAVI) was chosen to observe the soil background effect in old-growth forests as caused by the emergence of canopy gaps. As Sentinel-2A has the advantage of using the red-edge bands for vegetation monitoring (Frampton et al., 2013; Spracklen & Spracklen, 2019), we added red-edge related indices to utilize the red-edge capability in identifying old-growth forests. The Inverted Red-Edge Chlorophyll Index (IRECI) was chosen as it characterizes the red-edge slope and, at the same time, constitutes the maximum-minimum vegetation reflectance in the NIR and red bands (Frampton et al., 2013). Each VI was calculated in the raw band’s origin spatial resolution (10 m and or 20 m) and resampled to 30-m resolution afterward.

**Table 2:** The selected vegetation indices (VIs) were used in this study. All the bands used in the calculation had been resampled to 30-m resolution

Vegetation indices	Formula	Reference
NDVI	$\frac{B8-B4}{B8+B4}$	Rouse et al. (1974)
EVI	$\frac{2.5 \left(B8-B4\right)}{(B8+\left(6\times B4\right))-\left((7.5\times B2\right)+1)}$	Huete et al. (2002)
MSAVI	$\frac{2B8+1-\sqrt{{(2B8+1)}^{2}-8\left(B8-B4\right)}}{2}$	Qi et al. (1994)
IRECI	$\frac{B8-B4}{(B6/B5)}$	Frampton et al. (2013)

#### Textural feature

In addition to the spectral information, spatial information of multispectral images represented by textural variations may also enhance old-growth identification (Cohen & Spies, 1992). Textural variation describes a quantitative relationship of spectral values among the neighboring pixels and has shown in previous studies to improve forest stand classification (Coburn & Roberts, 2004; Cohen & Spies, 1992; Franklin et al., 2000; Meng et al., 2016; Spracklen & Spracklen, 2019; Zhang et al., 2017). Texture can be a viable proxy for assessing shadow emergence numbers in old-growth forests. Forest shadows reflect the uniformity and distribution of tree stands, and the greater complexity of mature forests leads to increased shadows and higher contrast due to diverse tree characteristics and reduced foliage cover from tree dieback (Cohen & Spies, 1992; Spracklen & Spracklen, 2019).

Grey-level co-occurrence matrix (GLCM) is the most common texture analysis method in remote sensing studies (Hall-Beyer, 2017; Haralick et al., 1973). The calculation of the GLCM is based on the variations of tone (represented by a digital number) between a pair of pixels. It considers a spatial relationship among all pixel pairs in the neighborhood (Hall-Beyer, 2017). Eight GLCM statistical measurements were used in this study to generate various textural features (Table 3). Each feature was measured based on a small moving window, i.e., 3×3 pixels, following the suggestion from Chen et al. (2004) that small window size is preferable to gain higher accuracy for spectrally homogenous classes at medium to coarse resolution imagery. As the targeted object for this study is forested areas, the spectral information of each class was relatively homogenous. The eight GLCM metrics were applied to each of the ten raw bands of Sentinel-2A with 10-m and 20-m resolutions, then resampled to 30-m resolution. The GLCM features generation was performed using ENVI 5.6.3 software.

**Table 3:** Description of GLCM texture metrics. *i* and *j* are row and column, respectively. In the construction of GLCM, *i* refers to the DN value of the target pixel, and *j* is the DN value of its neighbor. *P*(*i*,*j*) is the normalized co-occurrence matrix. *N* is the total number of pixels. *μ* and *σ* are the mean and standard deviation, respectively

GLCM texture features	Formula	Description
Mean (mean)	$\sum\limits_{i=1}^{{N}_{g}}.\sum\limits_{j=1}^{{N}_{g}}i*P(i,j)$	Measure the average value of the grey-level in the local kernel
Variance (var)	$\sum\limits_{i=1}^{{N}_{g}}.\sum\limits_{j=1}^{{N}_{g}}{(i-\mu )}^{2} P(i,j)$	Measures the grey-level distribution variations in an image
Homogeneity (hom)	$\sum\limits_{i=1}^{{N}_{g}}.\sum\limits_{j=1}^{{N}_{g}}\frac{1}{1+ {\left(i-j\right)}^{2}}P(i,j)$	Measures the closeness of the element in the GLCM to the GLCM diagonal
Contrast (con)	$\sum\limits_{i=1}^{{N}_{g}}.\sum\limits_{j=1}^{{N}_{g}}P(i,j){(i-j)}^{2}$	Measures the local variation in pixel values among the neighbors within an image
Dissimilarity (dis)	$\sum\limits_{i=1}^{{N}_{g}}.\sum\limits_{j=1}^{{N}_{g}}P\left(i,j\right)|i-j|$	Measures the differences in grey-scale level
Entropy (ent)	$\sum\limits_{i=1}^{{N}_{g}}.\sum\limits_{j=1}^{{N}_{g}}P(i,j) \text{log}(P\left(i,j\right))$	Measures the randomness of grey-level distribution
Second moment (sm)	$\sum\limits_{i=1}^{{N}_{g}}.\sum\limits_{j=1}^{{N}_{g}}{\{P\left(i,j\right)\}}^{2}$	Measures the uniformity or the number of repeated pixel pairs
Correlation (cor)	$\frac{{\Sigma }_{i}{\Sigma }_{j}\left(i,j\right)P\left(i,j\right)- {\mu }_{x}{\mu }_{y}}{{\sigma }_{x}{\sigma }_{y}}$	Measures the correlation between a pixel and its neighbor in the image

### Generating sample plots

Sample plots were generated randomly within the area of interest using the *create random points* toolbox in ArcGIS 10.8.2. There were 200 sample plots in total (100 plots for each old- and second-growth class) with a size of 0.1 ha generated from the BFNP stand age map polygons with a threshold of 150 years (> 150 years for old-growth and < 150 years for second-growth). A minimum distance of 300 m between the sample plots was set to reduce the effect of spatial autocorrelation. The stand age map was filtered by the land cover map of BFNP (Silveyra Gonzalez et al., 2018) to retain only the forested areas (Fig. 3) before it was used for generating the sample plots. This filtering step was taken to prevent generating random points in unwanted locations such as built-up areas, bare land/deadwood areas, meadows, shrubs, and clear-cut areas. The training and test datasets contained different stages of forest stand development (i.e., old- and second-growth) in two dominant species (i.e., spruce and beech) or mixed between them (Table 4). Seventy percent of the sample plots (140 plots) were assigned to train the classification, and the remaining 30% (60 plots) were used for testing the classifications. The train and test samples were randomly generated using the *caret* package in the R environment.

**Table 4:** The sample plot number of each class (i.e., old- and second-growth classes) per species

Class	Coniferous (Norway spruce)	Deciduous (European beech)	Mixed
Old-growth	46	20	34
Second-growth	36	29	35

### Classification and model assessment

Nineteen features from ALS and Sentinel-2A data were generated and divided into four groups, i.e., ALS feature, Sentinel-2A-raw band feature, Sentine-2A-vegetation indices (VIs) feature, and Sentinel-2A-texture feature (Table 5). We established three combination datasets for classification purposes (see Fig. 3).

**Table 5:** The ALS and Sentinel-2A features used in the classification. Collinear features were dropped through VIF analysis prior to the classification. After the VIF, 19 features of ALS and Sentinel-2A were selected for the classification. All features were resampled to 30-m resolution, adjusting the field plot size

Data	Features
ALS	Rumple Index; Vertical Complexity Index; zq85; zsd.
Sentinel-2A - Reflectance (raw bands)	Band 5 (Red-edge1 (703 nm)); Band 8 (Near-Infrared (842 nm)); Band 12 (Shortwave Infrared 2 (2190 nm)).
Sentinel-2A - Vegetation Indices	NDVI; EVI.
Sentinel-2A - Texture	B2_cor; B3_cor; B4_cor; B5_cor; B8_mean; B8_var; B8_hom; B8_cor; B11_cor; B12_cor.

The random forest algorithm (Breiman, 2001) was used to classify and estimate the importance of variables to optimize the model. For this study, two random forest parameters (number of trees (*ntree*) and input features (*mtry*)) were optimized following suggestions from Ayrey et al. (2019). Parameter optimization was applied to each dataset. We found that *ntree* of 1000 trees was the optimum setting for all datasets, while the optimum *mtry* was 3 to 5 depending on the dataset used in the classification. We performed a variable importance analysis using mean decrease accuracy (MDA) to select important features of each dataset. The most important features have to be selected for the model input to gain optimum classification performance (Shi et al., 2020). The number of selected features in the classification was determined by using different combinations of features from the MDA analysis in each dataset. The selection of combinations was generated by commencing a classification in a subset of the study area and gradually adding features from the top-ranked important features to the least. A* t*-test was conducted to evaluate the significance of the accuracy improvement (*p*-value < 0.05). The number of features and combinations were retained when the result achieved the highest accuracy.

Finally, to evaluate the accuracy of the old-growth maps generated from random forest, the classification accuracy of each dataset was assessed in a confusion matrix of 2 × 2 contingency table. User’s accuracy (UA), producer’s accuracy (PA), and F1-scores were used to assess the classification performance of the old-growth forest class per dataset. UA is the probability that a map pixel of a certain class corresponds to its class on the ground, while PA is the proportion of reference class pixels misclassified into other classes. F1-score is the harmonic mean of UA and PA, which is useful for dealing with unbalanced data (Kiala et al., 2019; Power, 2011). An additional assessment using the McNemar test (Mcnemar, 1947) was taken to observe the significant difference in the classification accuracy of each dataset. The classification, accuracy assessment, and variable importance analysis were performed in R software using the *randomForest* package (Liaw & Wiener, 2002).

## Results

### Classification performances from different datasets

The classification of each dataset, i.e., standalone Sentinel-2A, standalone ALS, and ALS + Sentinel-2A, was executed with the optimum number of input features after the random forest-MDA analysis. Overall, the ALS + Sentinel-2A combination obtained the highest classification accuracy for the old-growth class, followed by standalone ALS and multispectral, with an F1-score of 92%, 88%, and 71%, respectively (Table 6). The standalone Sentinel-2A and ALS + Sentinel-2A combination datasets exhibited a pattern: the old-growth class showed higher producer’s accuracy, while the second-growth class demonstrated higher user’s accuracy. In contrast to those two datasets, standalone ALS showed a slightly higher user’s accuracy in the old-growth class and higher producer’s accuracy in the second-growth class. These results demonstrated that there was a tendency of overestimation in old-growth prediction when multispectral (Sentinel-2A) features were used or added in the classification process. On the other hand, the standalone ALS was slightly better at identifying the actual old-growth forests on the ground.

**Table 6:** F1-score, producer’s accuracy (PA), user’s accuracy (UA) of old-growth forest class classification yielded by different datasets

Dataset	Old-growth class		
	PA (%)	UA (%)	F1-score (%)
Sentinel-2A	77%	66%	71%
ALS	87%	90%	88%
ALS + Sentinel-2A	93%	90%	92%

**Fig. 4:**
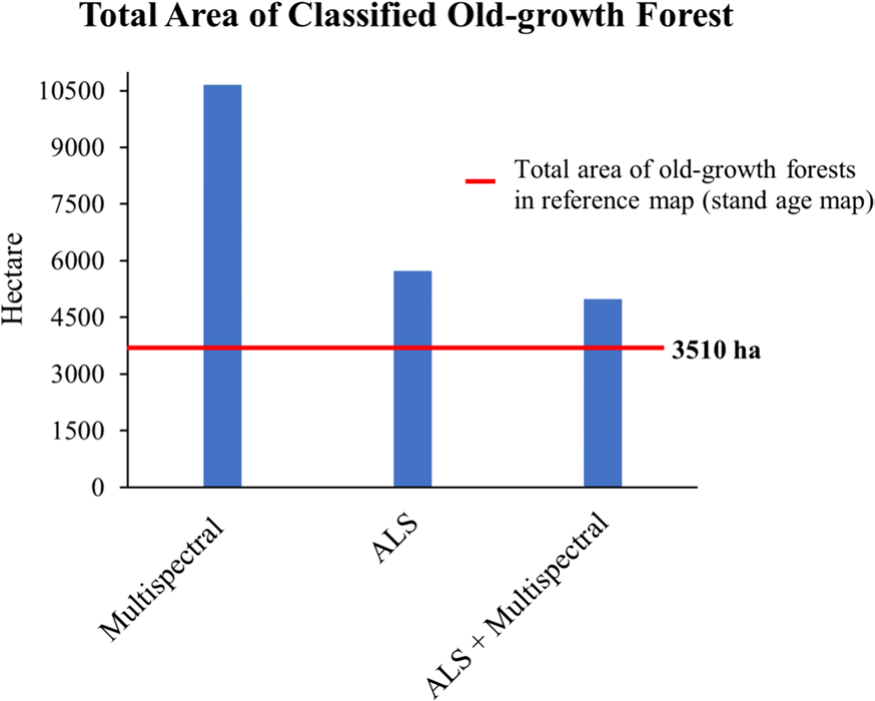
A comparison of total old-growth forest area per dataset resulted from the random forest classification. The improvement is shown when ALS was added to the classification, as demonstrated by the total area close to the red line (total area of old-growth forest in reference data (3510 ha))

**Fig. 5:**
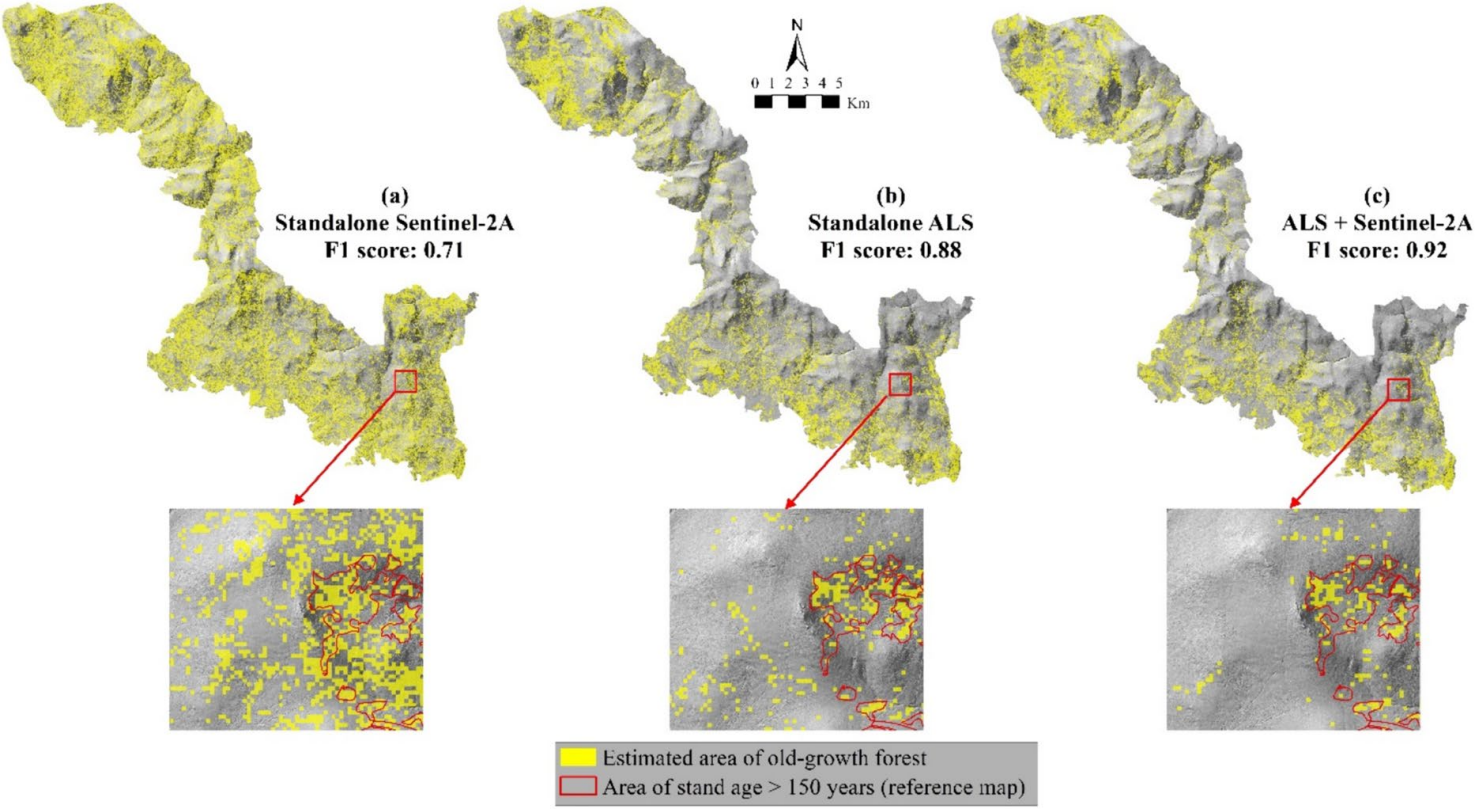
The results of the old-growth forests classification maps of each dataset. The inset of each map shows the improvements of overestimated old-growth forest polygons within an example area. It can be seen that from the standalone Sentinel-2A to the combination of ALS + Sentinel-2A, the grainy polygons are decreased and become more compact and clustered within the reference old-growth area. The grainy polygon decrease was also followed by increased F1-score of the old-growth forest class accuracy in each dataset classification. The combination of ALS and Sentinel-2A obtained the highest F1-score

The improvement in the accuracy was demonstrated by adding ALS data to the classification process. This was evidenced by the total area of classified old-growth forest that closely approximates the total area of the reference data (i.e., stand age map) (Fig. 4). As an example, the total area of the old-growth forest of the datasets containing ALS data decreased drastically compared to the Sentinel-2A feature datasets. The grainy polygons of the estimated old-growth class also decreased immensely in datasets that contained ALS data (Fig. 5). It can be implied that the Sentinel-2A data overclassified the old-growth forest class compared to the ALS-contained datasets.

The significant difference between the classification results is shown by the *p*-value of McNemar’s test for each pair dataset classification (Table 7). The accuracy of the old-growth class using standalone ALS (88%) and ALS + Sentinel-2A combination (92%) datasets significantly outperformed standalone Sentinel-2A (71%). However, the classification accuracy of standalone ALS is comparable to the combination of the ALS + Sentinel-2A dataset.

**Table 7:** McNemar test for pairwise comparison of each dataset classification. *, *p* < 0.05; ns, *p* > 0.05

	Sentinel-2A	ALS	ALS + Sentinel-2A
Sentinel-2A	-	*	*
ALS		-	ns

### Feature importance in the classification

**Fig. 6:**
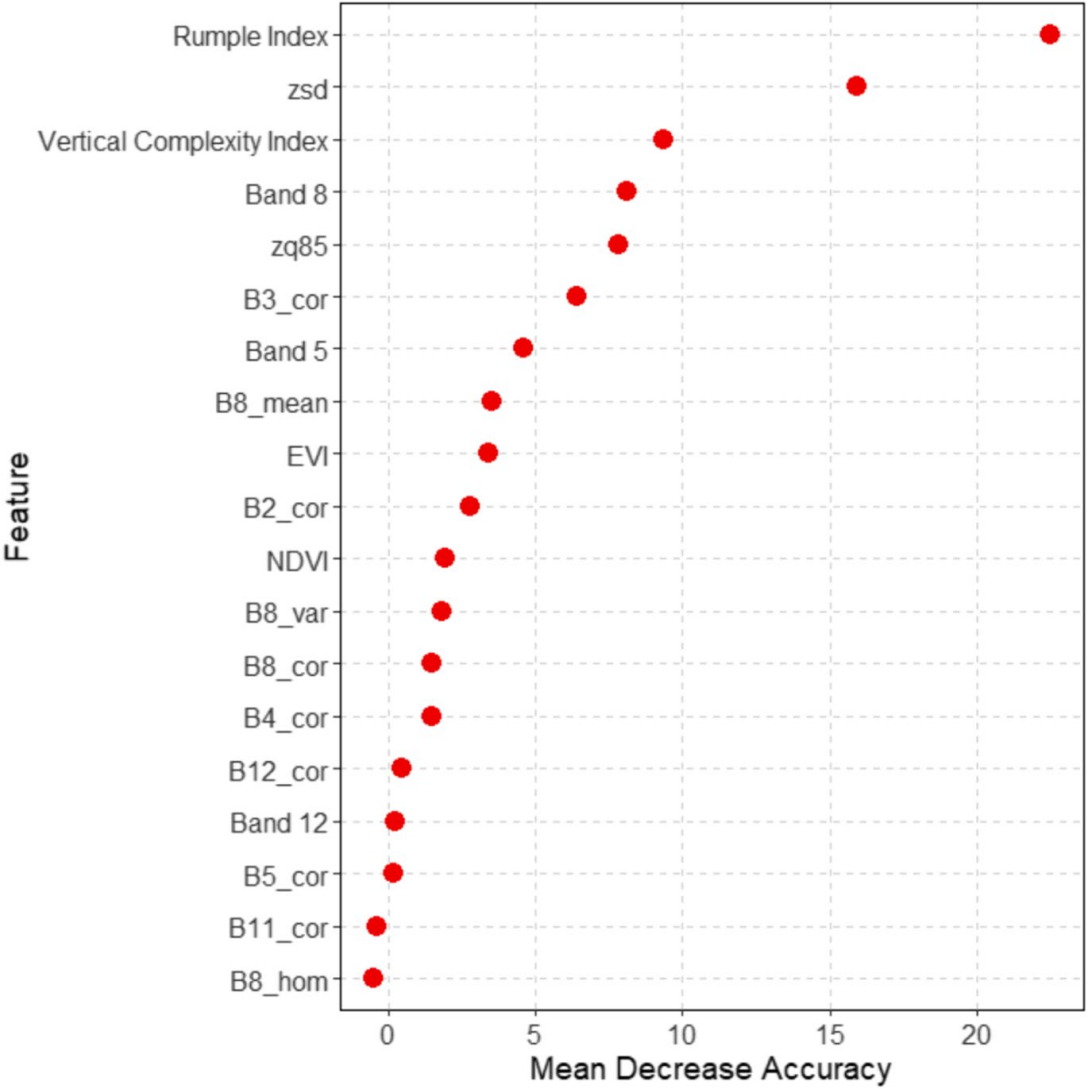
The variable importance of all ALS and Sentinel-2A features generated from mean decrease accuracy (MDA) analysis. These features were used in the classification using an ALS + Sentinel-2A combination dataset

We ranked all features of ALS and Sentinel-2A features (raw band, VIs, and texture) datasets based on the mean decrease accuracy (MDA) result to observe their relative importance in the classification (Fig. 6). All ALS features, i.e., Rumple Index, zsd, zq85, and Vertical Complexity Index (VCI), were in the top five ranks of the important features. Only one Sentinel-2A feature, i.e., Band 8 (near-infrared) is placed in the top five important features. These top five important features demonstrated a substantially higher mean decrease accuracy (MDA) score than other features. The ranking pattern of each standalone ALS and Sentinel-2A dataset is similar to the MDA ranking of the combination dataset (see Fig. S1a and b in the supplementary). It was shown that vegetation indices (EVI and NDVI) were not as important in the old-growth classification compared to other Sentinel-2A features (i.e., reflectance/raw bands and texture).

## Discussion

Protecting the remaining old-growth forests is a part of the 2030 EU Biodiversity Strategy. Therefore, developing an approach for identifying and mapping the distribution of old-growth forests across Europe using remote sensing is essential (Hirschmugl et al., 2023). This study investigates the application of airborne LiDAR (ALS) and Sentinel-2A multispectral data for mapping old-growth forests in Central European mixed temperate forests in BFNP, Germany. Our study utilized comprehensive horizontal and vertical structure complexity information to map old-growth forest distribution by combining spectral and structural information from Sentinel-2A and ALS, respectively, to improve the mapping accuracy. The combination of ALS and Sentinel-2A datasets yielded the highest classification accuracy compared to standalone Sentinel-2A and standalone ALS datasets, with an F1-score of 92% and producer’s and user’s accuracies of 93% and 90% for the old-growth class, respectively.

### Performance of the ALS and Sentinel-2A in the old-growth mapping

The results demonstrated that structural information comprising horizontal-vertical profiles from ALS was essential in distinguishing old-growth forests. When the ALS data was added to the Sentinel-2A data, the F1-score of old-growth forest class was significantly improved by 20%. Further, standalone ALS showed 17% higher F1-score than standalone Sentinel-2A. The accuracies between standalone ALS and the combination of ALS + Sentinel-2A showed insignificant differences (Table 7), with only a 4% increase.

We found that using or adding the Sentinel-2A dataset overestimated the identified old-growth forests, as shown by the higher producer’s than the user’s accuracy (Table 6). It was assumed that the overestimation was due to the lack of capability of Sentinel-2A to differentiate the detailed structural complexity, mainly the canopy layering (Scarth et al., 2001). Another cause of the overestimation could be that the top canopy of old- and second-growth classes shared a similar reflectance as mature stands dominating the BFNP landscape. As our second-growth samples were dominated by stand ages between 80 and < 150 years (which are considered mature stands), this confusion with the old-growth stand (> 150 years) top canopy is possibly due to the developed canopy within the second-growth stage.

The ALS data showed its capability by providing structural complexity information that significantly improved the accuracy of the old-growth mapping compared to standalone Sentinel-2A (see Tables 6 and 7). Standalone ALS and the combination of ALS + Sentinel-2A accurately captured the total area of old-growth forests in the reference dataset (i.e., BFNP stand age map), compared to standalone Sentinel-2A. Our study also confirmed the results from Lahssini et al. (2022), where forest structure identification was more accurately derived when Sentinel-2A is combined with ALS. Besides the improvement, a combination of ALS and Sentinel-2A data has an advantage in improving the range of identified biophysical indicators (Scarth et al., 2001). The broader range of biophysical indicators is essential for old-growth forest identification, as the derived information will integrate horizontal and vertical canopy profiles to define the complexity of the old-growth stage. Additionally, the combination of ALS + Sentinel-2A also reduced the grainy appearance of the polygons of the estimated old-growth forest to be more compact (Fig. 4). This may indicate that the combined sensors may improve the classification by reducing the overestimation from the standalone Sentinel-2A.

### Important features for identifying the old-growth forests

Our study results suggested that remote sensing features sensitive to structural information were more essential than spectral information when identifying the old-growth stage. It follows that relying only on spectral information may not be sufficient in old-growth classification, especially in a landscape dominated by mature canopy forests, as there is a lack of spectral variations in the top canopy (Coburn & Roberts, 2004). Further, the top three important Sentinel-2A features (see Fig. S1b in the supplementary) consisted of red-edge band (Band 5), near-infrared (Band 8), and texture (B3_correlation), which are sensitive to forest structure. Red-edge/near-infrared bands and textural information have a common characteristic; they indicate the emergence and proportion of forest shadows (Spracklen & Spracklen, 2019; Xu et al., 2019).

Concerning the importance of variables for old-growth classification, we found that vegetation indices (VIs) were less important than reflectance and textural features in the Sentinel-2A dataset. This was shown by the variable importance rank, where VIs were not in the top five features of important Sentinel-2A features (see Fig. S1b in the supplementary). Our findings aligned with a study from Spracklen and Spracklen (2019), where VIs insignificantly improved the accuracy of old-growth forest classification. We presume that the less important VIs in old-growth forest classification were caused by saturation in a dense canopy. As mature forests predominate the BFNP landscape, the overstory canopies of second-growth are relatively dense, and this limits the VIs in characterizing surface vegetation, which is potentially confused with large canopies in old-growth areas (Gao et al., 2023). Nevertheless, VIs may be useful in the pre-classification process to mask forest and non-forest areas and could be important in discriminating old-growth stages based on tree species composition (Spracklen & Spracklen, 2019), which was not investigated in our study.

The occurrence of mortality events that create canopy complexity in the old-growth stage revealed an interesting finding regarding forest shadow. Mortality events in older forests potentially generate an irregular horizontal distribution of structures, leading to structural complexity (Franklin & Van Pelt, 2004). This complexity becomes pronounced with an increase of large canopy gaps, heterogeneous canopy sizes, and different tree heights (Cohen & Spies, 1992; Fiorella & Ripple, 1993; Franklin & Van Pelt, 2004). Further, these structural change characteristics cause a decrease in reflected light in the canopy, creating a greater proportion of shadows (Spracklen & Spracklen, 2019). Commonly, younger forests have less structural complexity due to a more uniform spatial distribution, equal tree heights, and uniform canopy size (Spracklen & Spracklen, 2019). These characteristics of earlier stages create denser and continuous canopies, resulting in few or even no forest shadows.

Our study also confirmed the studies of Spracklen and Spracklen (2019) and Zhang et al. (2017) that texture is essential in differentiating forest stand development, particularly for identifying the complexity of old-growth forests. Old-growth forests have a higher contrast textural value due to the complexity of the forest structure compared to the second-growth. Second-growth forests commonly have a low contrast textural value as the overstory canopies are relatively more uniform and denser. The complexity of the old-growth structure is mainly caused by mortality occurrences such as self-thinning, dieback trees, and canopy gaps (Franklin & Van Pelt, 2004). Image texture can also indicate the emergence of shadow as the surface roughness is associated with the various heights of the trees (Zhang et al., 2017). The higher contrast of textural value in multispectral data may indicate that the old-growth stage has more varied tree heights and gaps, leading to a greater proportion of forest shadows. Similar to the textural feature in multispectral imagery, the ALS feature, i.e., the Rumple Index, can model the proportion of forest shadows (Kane et al., 2008). The higher value of the Rumple Index indicates an old-growth stage (Chamberlain et al., 2021) as it represents more complex spatial heterogeneity of the canopy and gap sizes in an area and further suggests a higher proportion of forest shadows (Kane et al., 2008). Our findings suggested forest shadows as one of the old-growth indicators by incorporating textural and reflectance at red-edge and near-infrared wavelengths and the Rumple Index from ALS. Although shadows are often considered noise in image classification, they are suitable for indicating the complexity and heterogeneity of old-growth forest structures (Cohen & Spies, 1992).

### Limitations and outlook for future studies and forest management

It is worth noting that our reference data (i.e., the stand age map) was generated from the interpolation and generalization of inventory plots. This may produce uncertainties about on-the-ground conditions between the plots. Therefore, past researchers have suggested combining different remotely sensed data, particularly high-resolution imagery, to minimize uncertainties and fill the information gap (Scarth et al., 2001). Using or adding ALS in the analysis may be one of the alternatives to dealing with interpolation-based reference data, as shown by the improvements in the mapping accuracy in this study.

It is important to note that exploratory analyses revealed that areas defined as old-growth forests but classified as second-growth by the algorithm were often dominated or mixed with broadleaf tree species (see Table S2-C and D in the supplementary). We presumed the misclassification was caused by (1) the relative uniformity of horizontal heterogeneity in the upper canopy, (2) a small number and size of canopy gaps, and (3) a denser canopy in the older stand. Those characteristics are similar to the upper canopy of second-growth forests. The uniformity might underestimate the value of the Rumple Index in the ALS dataset, and the denser canopy creates fewer shadows that may have gone undetected by image texture. The lack of old-growth broadleaf forest samples compared to conifer and mixed forests (see Table 4) may also cause misclassification. With more broadleaf samples, VIs could be important, especially in identifying the composition of tree species in old-growth forests (Spracklen & Spracklen, 2019).

A suggestion to overcome this issue is to include understory configurations in the classification. This can be challenging, especially in detecting detailed vertical structures and understory dense broadleaf stands, which can be difficult for ALS to capture. One approach to address this drawback is to use a close-range sensing technology, such as a terrestrial laser scanner (TLS). Another approach is to utilize a low-altitude flight acquisition using an uncrewed aerial vehicle (UAV)-LiDAR with high point cloud density to ensure the detection of objects below the upper canopy. Hamraz et al. (2017) found that a minimum point cloud density of 170 points/m^2^ of UAV-LiDAR can penetrate the upper canopy, reduce the occlusion of foliage cover, and thus improve the identification of understory objects. However, TLS and UAV-LiDAR were developed to capture detailed information at a plot level with smaller coverage. They encounter challenges similar to ALS in mapping large areas because they are costly and labor-intensive.

The findings from this study contribute to developing an old-growth forest mapping approach within the Central European mixed temperate forests ecoregion. Our approach showed that using a combination of different types of remote sensing sensors has the potential to be applied for mapping old-growth forests in other temperate forest regions across Europe. However, it should be noted that the interpolation of our models can be done only in landscapes with similar growing conditions, species composition, the trajectory of disturbances, and forest management compared to our study site in BFNP. Those conditions must be considered as this ecoregion spans 7000 km^2^ across Central Europe and covers ten countries (Olson et al., 2001). Each country may have a different protection policy, forest management, and even definition of old-growth forests (O’Brien et al., 2021). To use our model for old-growth mapping across European temperate forests, splitting the mapping area based on geographical criteria (e.g., country or biome) to accommodate specific definitions, disturbance drivers, and threshold values may be necessary (Hirschmugl et al., 2023). It should also be noted that to apply our model to different forest types, using a definition of old-growth forests based on the biomes and ecosystems where they exist is required since ecosystem structure and disturbance drivers may vary between biomes (Tíscar & Lucas-Borja, 2016).

Managers can apply our integrated approach and map to assess and locate areas that potentially have characteristics of old-growth forests. By locating the potential areas, managers will be better equipped to inventory and develop management plans to conserve these areas. This mapping approach can be integrated into forest management plans to facilitate forest area monitoring. Regarding capturing high-resolution forest structure data for monitoring activities, stakeholders (e.g., managers and governmental agencies) should consider combining free satellite multispectral imagery (e.g., Sentinel-2A and Landsat) with structure-sensitive sensors, such as ALS. Although satellite multispectral is capable of covering large areas with denser temporal acquisition, it has a downside in delivering detailed forest structures, especially the structures that are related to vertical layering (Lahssini et al., 2022; Spracklen & Spracklen, 2019). Therefore, ALS acquisitions should be prioritized to provide structural information on old-growth forests. Deployments of ALS-based mapping have the potential to provide detailed 3D forest information in the broader area when flown more extensively. Another alternative to obtain structural information in wide areas is to substitute ALS with satellite-based remote sensing sensors sensitive to forest structure, such as GEDI (LiDAR) and Sentinel-1 (Radar). Both GEDI and Sentinel-1 are publicly accessible and open opportunities to be combined with satellite multispectral data. However, further study is needed to investigate the capability of the combination sensors in classifying old-growth forests, especially with GEDI, as it is a sampling concept mission.

## Conclusion

This study optimized the mapping of temperate old-growth forests in Central European mixed forests by combining airborne laser scanning (ALS) and Sentinel-2A. The combination was performed to overcome the limitation of multispectral (represented by Sentinel-2A imagery) in providing 3D forest structural complexity information of old-growth forests to a larger extent. It was demonstrated that adding ALS data can significantly improve the old-growth classification with an increase of 20% in the classification accuracy. This finding indicates that structural characteristics are essential for classifying old-growth forests. The old-growth stage is structurally complex and characterized by various canopy sizes, tree heights, and a large number of gaps. These variations also lead to the emergence of forest shadows, which can be a suitable indicator for old-growth forest identification, besides the complexity of the horizontal and vertical canopy. Therefore, using remote sensing sensors and features sensitive to forest structures, such as ALS and textural features with reflectance at red-edge and near-infrared wavelengths for multispectral, is recommended. Forest managers and decision-makers can benefit from this approach to decide optimal remote sensing sensors and features for old-growth forest detection, particularly when incorporating open public remote sensing datasets into routine forest monitoring workflows.

## Supplementary Information

Below is the link to the electronic supplementary material.

Supplementary Material

## Data Availability

All the datasets used in this study are available upon request to the corresponding author.
