# Supplementary material for: Mapping temperate old-growth forests in Central Europe using ALS and Sentinel-2A multispectral data
Source: Environ Monit Assess. 2024 Aug 26;196(9):841. doi: 10.1007/s10661-024-12993-5 (PMC11345331; doi:10.1007/s10661-024-12993-5)
Supplement: Supplementary file 1 — Supplementary Material [file 10661_2024_12993_MOESM1_ESM.pdf]

SUPPLEMENTARY MATERIAL

Mapping temperate old-growth forests in Central Europe using ALS and Sentinel-2 multispectral data

This file includes:

- Tables S1-S2
- Figure S1

Table S1. The description of ALS features that were generated for this study. There were 61 features generated from point cloud and canopy height model (CHM). The features were divided into two groups, i.e., standard features (54 features) and structural features (7 features). Based on multicollinearity analysis, only 4 features with VIF < 5, i.e., rumple index, vertical complexity index, zsd and zq85. Thus, these features were used later in the classification.

	Features	Description	Category Description
**Standard Features**			
	ZMAX	Maximum height	Height-related/vertical profile of the point clouds
	ZMEAN	Mean height above ground	
	ZSD	Standard deviation of height distribution	
	ZSKEW	Skewness of height distribution	
	ZKURT	Kurtosis of height distribution	
	ZENTROPY	Entropy of height distribution	
	ZQx	x^{th} percentile of height distribution (5,10, 15,20,…...,99)	
	PZABOVE2	Returns percentage above 2 m	Number of returns/density based on the point clouds
	PZABOVEZMEAN	Returns percentage in above mean height	
	ZPCUMx	Cumulative percentage of return in the xth layer (1 to 9)	
	Pxth	Percentage in x returns	
	PGROUND	Returns percentage classified as "ground."	
**Structural Features**			
**Canopy Height Model**	Rumple	Ratio between outer canopy surface area and projected ground surface	These attributes are related to canopy roughness, which describes the structural heterogeneity as an impact of distribution and various sizes of canopy gaps and various tree heights (Hardiman *et al*., 2011; Kane *et al*., 2010b)
	Rugosity	An outer canopy roughness measured by standard deviation of CHM	
	Deep Gaps Fraction	Fraction of canopy gaps per square meter	These attributes are related to the distributions of canopy gaps and openness estimation (Atkins *et al*., 2018)
	Cover Fraction	The inverse of the deep gap fraction	
	Gaps Fraction	Distribution of gaps in the canopy volume	
**Vertical Distributio n**	Vegetation Area Index (VAI)	sum of leaf area density within the canopy volume	VAI calculates the area, density, and volume of vegetation, which is derived from the sum of leaf area density (LAD)-a divided leaf area index (LAI) within height interval (Atkins *et al*., 2018; Bouvier *et al*., 2015; Kamoske *et al*., 2019; Lefsky *et al*., 1999)
	Vertical Complexity Index (VCI)	The distribution evenness of point cloud within a vertical layer	VCI is based on the information theory index used for quantifying species evenness. It can also quantify the evenness of three-dimensional point cloud distribution within a vertical layer (van Ewijk *et al*., 2011)

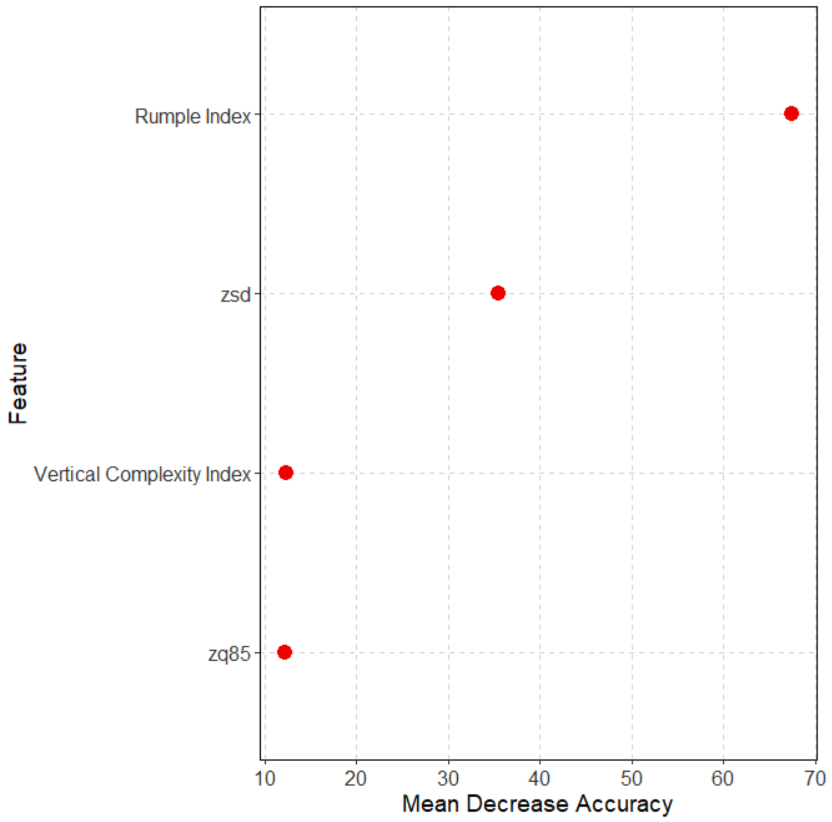

(a)

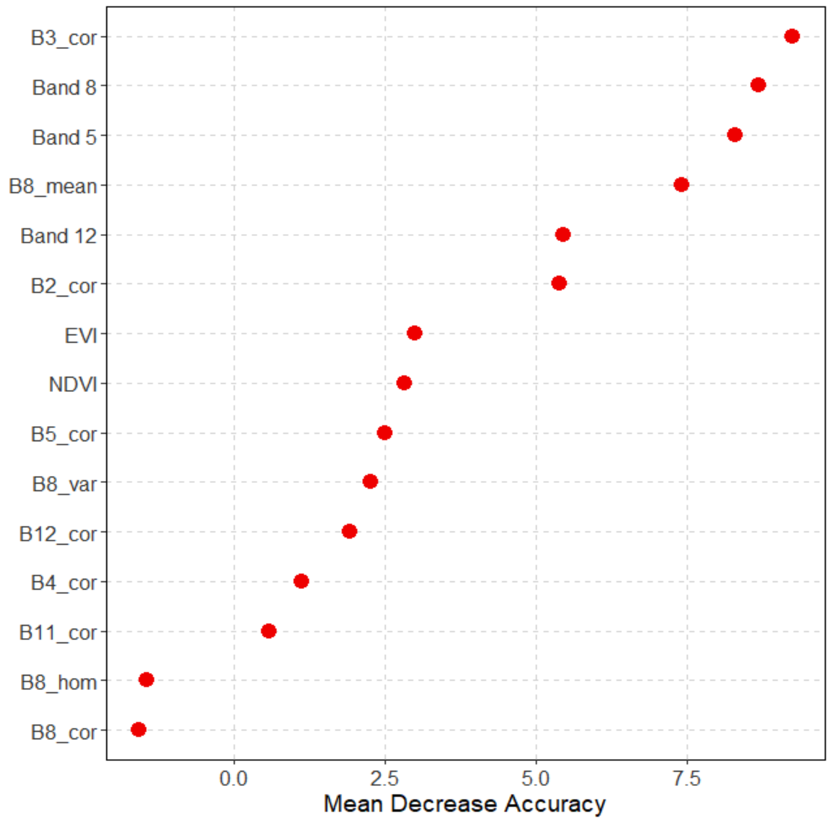

(b)

Fig. S1. The variable importance ranking of ALS and Sentinel-2A datasets. The ranking was generated from Random Forest Mean Decrease Accuracy (MDA) analysis. a) ALS features; b) Sentinel-2A features (raw bands, vegetation indices, and texture) All the features have been selected through multicollinearity analysis using variance inflation factor (VI) ≤ 5.

Table S2. Examples of the old-growth forest classification result (the correctly classified and misclassified). We compared the classification map, aerial photo, and ground photo to depict the actual ground situation. The red dot is the center point of the field plot. The areas with yellow shading are the classified old-growth produced by the Random Forest algorithm, and the areas without shading are second-growth. The aerial photograph was acquired on June 11, 2017. The presented classification maps are from the highest classification accuracy dataset, i.e., a combination of ALS and Sentinel-2A, with F1-score of 92% for old-growth class.

Plot Location	Classification Map	Aerial Photo (res. 10 cm)	Ground Situation	Status
**A** 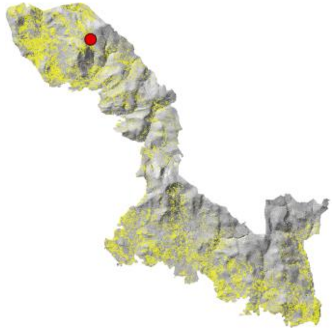	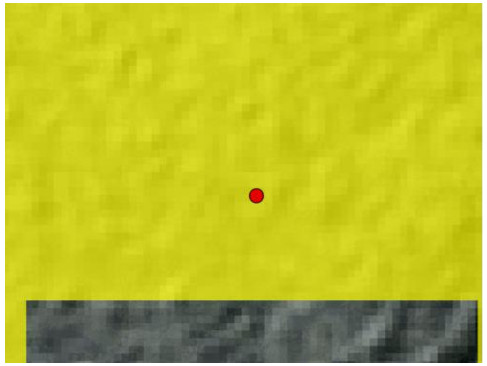	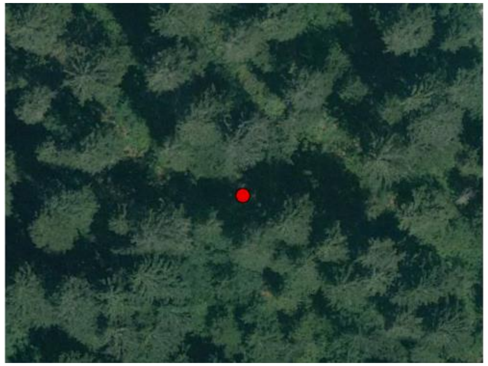	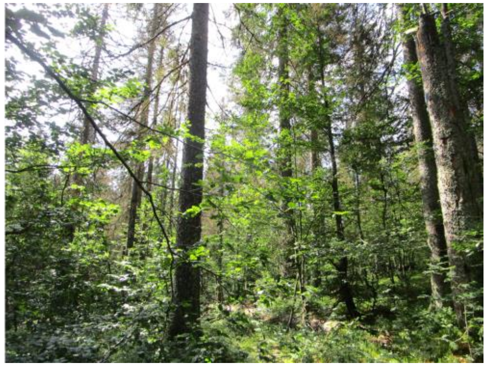	Correctly classified (old-growth class)
**B** 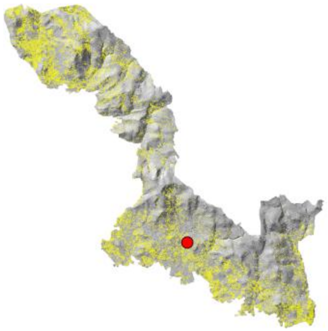	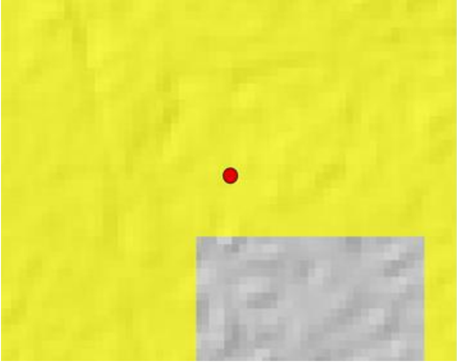	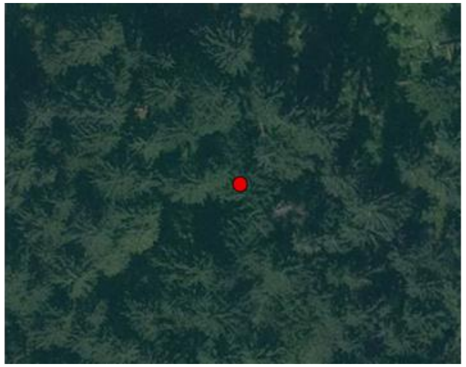	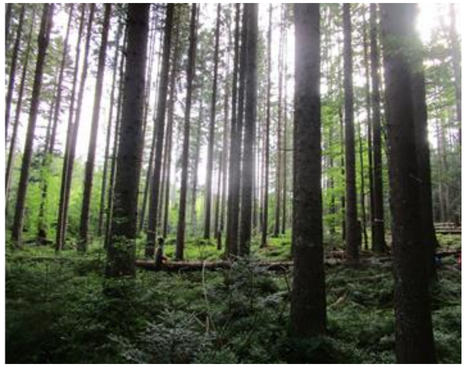	Correctly classified (old-growth class)

**C** 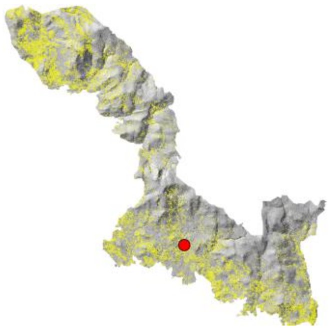	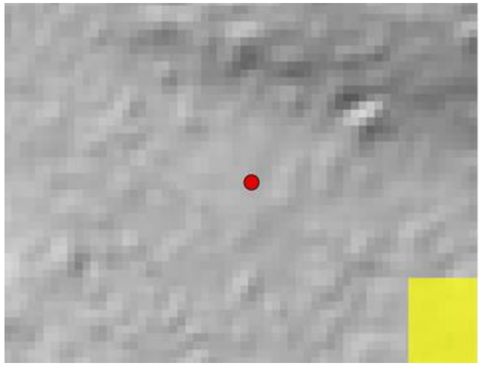 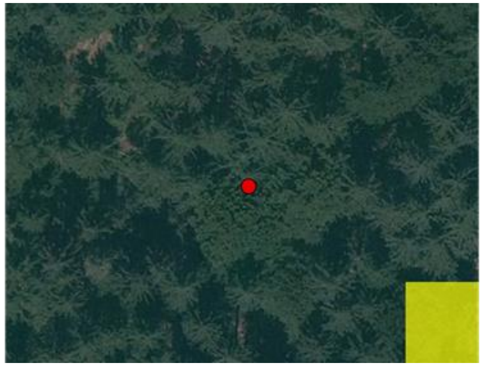 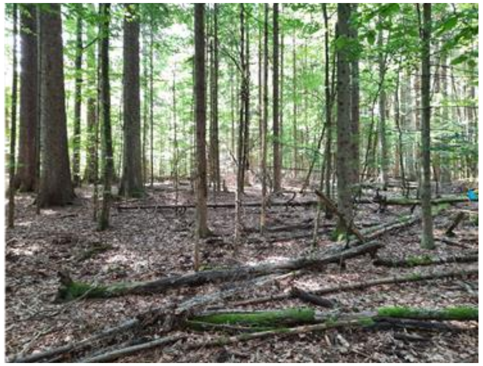	Misclassified (Defined as old-growth, classified as second-growth)
**D** 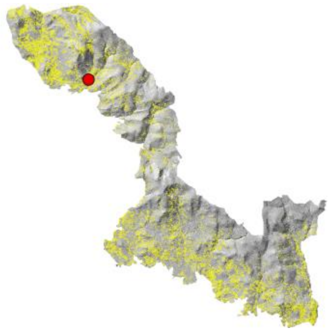	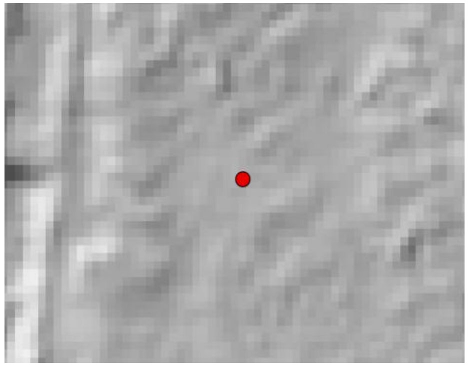 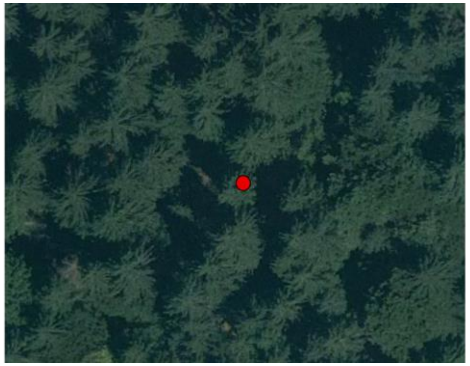 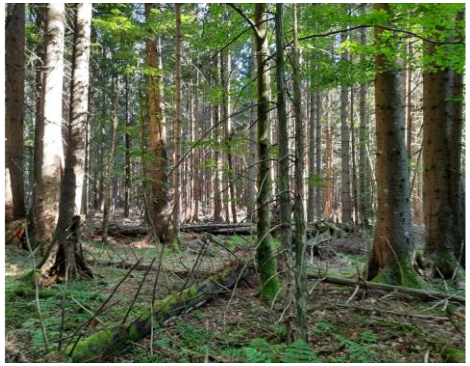	Misclassified (Defined as old-growth, classified as second-growth)

Reference

Atkins, J. W., Fahey, R. T., Hardiman, B. H., & Gough, C. M. (2018). Forest Canopy Structural Complexity and Light Absorption Relationships at the Subcontinental Scale. *Journal of Geophysical Research: Biogeosciences*, *123*, 1387–1405. https://doi.org/10.1002/2017JG004256

Bouvier, M., Durrieu, S., Fournier, R. A., & Renaud, J. P. (2015). Generalizing predictive models of forest inventory attributes using an area-based approach with airborne LiDAR data. *Remote Sensing of Environment*, *156*, 322–334. https://doi.org/10.1016/j.rse.2014.10.004

Hardiman, B. S., Bohrer, G., Gough, C. M., Vogel, C. S., & Curtis, P. S. (2011). The role of canopy structural complexity in wood net primary production of a maturing northern deciduous forest. *Ecology*, *92*(9), 1818–1827. https://doi.org/10.1890/10-2192.1

Kamoske, A. G., Dahlin, K. M., Stark, S. C., & Serbin, S. P. (2019). Leaf area density from airborne LiDAR: Comparing sensors and resolutions in a temperate broadleaf forest ecosystem. *Forest Ecology and Management*, *433*, 364–375. https://doi.org/10.1016/j.foreco.2018.11.017

Kane, V. R., McGaughey, R. J., Bakker, J. D., Gersonde, R. F., Lutz, J. A., & Franklin, J. F. (2010). Comparisons between field- and LiDAR-based measures of stand structural complexity. *Canadian Journal of Forest Research*, *40*, 761–773. https://doi.org/10.1139/X10-024

Lefsky, M. A., Cohen, W. B., Acker, S. A., Parker, G. G., Spies, T. A., & Harding, D. (1999). Lidar remote sensing of the canopy structure and biophysical properties of Douglas-fir western hemlock forests. *Remote Sensing of Environment*, *70*, 339–361. https://doi.org/10.1016/S0034-4257(99)00052-8

van Ewijk, K. Y., Treitz, P. M., & Scott, N. A. (2011). Characterizing forest succession in central Ontario using lidar-derived indices. *Photogrammetric Engineering and Remote Sensing*, *77*(3), 261–269. https://doi.org/10.14358/PERS.77.3.261
